# TREM2 mediates physical exercise-promoted neural functional recovery in rats with ischemic stroke via microglia-promoted white matter repair

**DOI:** 10.1186/s12974-023-02741-w

**Published:** 2023-02-25

**Authors:** Jinghui Xu, Liying Zhang, Mingyue Li, Xiaofei He, Jing Luo, Rui Wu, Zhongqiu Hong, Haiqing Zheng, Xiquan Hu

**Affiliations:** Department of Rehabilitation Medicine, The Third Affiliated Hospital, Sun Yat-Sen University, No. 600 Tianhe Road, Guangzhou, China

**Keywords:** Ischemic stroke, Physical exercise, Triggering receptor expressed on myeloid cells 2, Brain white matter repair, Microglia

## Abstract

**Background:**

The repair of white matter injury is of significant importance for functional recovery after ischemic stroke, and the up-regulation of triggering receptors expressed on myeloid cells 2 (TREM2) after ischemic stroke is neuroprotective and implicated in remyelination. However, the lack of effective therapies calls for the need to investigate the regenerative process of remyelination and the role of rehabilitation therapy. This study sought to investigate whether and how moderate physical exercise (PE) promotes oligodendrogenesis and remyelination in rats with transient middle cerebral artery occlusion (tMCAO).

**Methods:**

Male Sprague–Dawley rats (weighing 250–280 g) were subjected to tMCAO. AAV-shRNA was injected into the lateral ventricle to silence the *Trem2* gene before the operation. The rats in the physical exercise group started electric running cage training at 48 h after the operation. The Morris water maze and novel object recognition test were used to evaluate cognitive function. Luxol fast blue staining, diffusion tensor imaging, and electron microscopy were used to observe myelin injury and repair. Immunofluorescence staining was applied to observe the proliferation and differentiation of oligodendrocyte precursor cells (OPCs). Expression of key molecules were detected using immunofluorescence staining, quantitative real-time polymerase chain reaction, Western blotting, and Enzyme-linked immunosorbent assay, respectively.

**Results:**

PE exerted neuroprotective efects by modulating microglial state, promoting remyelination and recovery of neurological function of rats over 35 d after stroke, while silencing *Trem2* expression in rats suppressed the aforementioned effects promoted by PE. In addition, by leveraging the activin-A neutralizing antibody, we found a direct beneficial effect of PE on microglia-derived activin-A and its subsequent role on oligodendrocyte differentiation and remyelination mediated by the activin-A/Acvr axis.

**Conclusions:**

The present study reveals a novel regenerative role of PE in white matter injury after stroke, which is mediated by upregulation of TREM2 and microglia-derived factor for oligodendrocytes regeneration. PE is an effective therapeutic approach for improving white matter integrity and alleviating neurological function deficits after ischemic stroke.

**Supplementary Information:**

The online version contains supplementary material available at 10.1186/s12974-023-02741-w.

## Introduction

Emerging evidence shows that long-term neurological function deficits after stroke may be partly attributed to white matter damage [1], except for the neuron loss within gray matter. White matter comprises axons, oligodendrocytes, microglia, and astrocytes [2]. Multiple mechanisms, including axon degeneration, demyelination, and neuroinflammation are implicated in white matter injury after stroke [2]. Notably, remyelination is a central part of white matter repair with novel generated myelin sheath in the adult central nervous system after injuries [2]. Remyelination relies on novel, mature oligodendrocytes [3–5]. Although proliferation and migration of oligodendrocyte precursor cells (OPCs) are improved after stroke, most OPCs fail to differentiate into mature oligodendrocytes primarily due to deleterious environment in the lesioned brain, thus impeding the remyelination and neurological function recovery after stroke [2]. Thus, therapeutic interventions improving the deleterious environment in the lesioned brain are beneficial for differentiating OPCs into mature oligodendrocytes and axonal remyelination after stroke.

Several studies have confirmed that physical exercise (PE) promotes neurological function recovery after stroke [6–8]. Numerous mechanisms, including up-regulated expression of neurotrophic factors or proliferation of nerve cells, improved angiogenesis or synaptic plasticity, and white matter integrity, are implicated in the role of PE-promoted recovery after stroke [9]. However, the underlying mechanism remains unclear despite emerging evidence showing that PE could promote oligodendrocyte lineage development and myelination [10]. Our previous studies demonstrated that PE could promote the recovery of chronic cerebral hypoperfusion (CCH)-induced cognitive impairment by shifting microglia and astrocyte towards a neuroprotective phenotype, thereby creating a more beneficial microenvironment for oligodendrocyte genesis and white matter repair [10, 11]. The mechanisms by which PE promotes white matter injury repair and neurological function recovery after ischemic stroke should be further investigated since the local white matter injury is more severe in ischemic stroke than in chronic cerebral hypoperfusion. It should be noted that, though previous studies [12] have claimed different phenotypes of activated microglia after brain injury according to expression spectrum of pro- and anti-inflammatory factors and its functions: classically activation or alternatively activation phenotype, more and more recent studies indicated overlapping of these two subtypes and pointed out that microglial complexity could not be reduced to oversimplified [13]. Therefore, the role and mechanisms through which PE regulates microglial state and function remain to be investigated.

Recent studies have shown that triggering receptors expressed on myeloid cells 2 (TREM2) is necessary for remyelination [14], and disruption of the *TREM2/DAP12* pathway induces neurodegeneration with demyelination and axonal loss [15]. However, the mechanism of TREM2-promoted remyelination remains unclear, though TREM2 up-regulation after ischemic stroke is protective for neurological function [15]. Moreover, TREM2 is primarily expressed in microglia, activated macrophages. It could modulate microglial state, improve the environment in the lesioned brain, as well as promote white matter integrity [15, 16]. Besides, Jensen et al. indicated that PE-promoted TREM2 up-regulation in cerebral spinal fluid might benefit Alzheimer’s disease patients [17]. However, the effect of PE on TREM2 expression after stroke remains unreported. Therefore, it is necessary to investigate whether PE modulates micrlglial state and promotes remyelination after stroke via TREM2.

Miron et al. showed that microglia-derived activin-A, a member of the TGF-β superfamily, is vital for the differentiation of OPCs towards mature oligodendrocytes and activin-A inhibition could suppress microglia-derived remyelination [18]. Activin-A exerts its effect through binding its receptors (Acvr 2b, 2a, etc.). Previous studies also demonstrated the neuroprotective properties of activin-A and the upregulation of its receptors on OPCs in remyelinating lesions, indicating it may be a promising novel therapeutic target for remyelination [18]. However, whether PE promotes microglia-derived activin-A remains unknown. Herein, we aimed to investigate the effect of PE on TREM2 expression and its potential roles in white matter repair and neurological function recovery in rats with transient middle cerebral artery occlusion (tMCAO).

## Methods

### Rat models of transient cerebral ischemia

Maximum efforts were made to minimize the number of animals sacrificed and the suffering of animals. All animals were housed in a standard experimental animal room, with controlled temperature and humidity, as well as a 12-h light/dark cycle. All animals were free access to water and food. A total of 175 eight weeks old male Sprague–Dawley (SD) wide-type rats weighing 250–280 g were purchased from Beijing Vital River Laboratories Co., Ltd. (Beijing, China). A total of 29 rats that died after ischemia and six rats that failed to induce ischemia were excluded.

Animals were randomly assigned to sham group or transient middle cerebral artery occlusion (tMCAO) groups. The operation was performed by intraluminal occlusion at the right middle cerebral artery (MCA) for 1 h. Sham-operated animals underwent similar exposure of arteries without middle cerebral artery occlusion. The operation was performed by one investigator blinded to the experimental grouping as previously described [19]. Briefly, the rats were anesthetized with 1% pentobarbital until they were unresponsive in the tail-pinch test. During surgery, rectal temperature was maintained at 37.0 ± 0.5 °C using a temperature-regulated heating pad.

### Preparation of Adeno-associated virus (AAV) particles

AAV particles were purchased from Keygen Biotech. Co. Ltd. (Nanjing, China). Short hairpin sequences were synthesized (TREM2-shRNA: Gene ID: NM_001106884.1 5′-GATCCGCCAATCAGGAAAGACCTTCTCTCGAGAGAAGGTCTTTCCTGATTGGCTTTTTA-3′ and vehicle-shRNA: 5′-GATCCTCGCTTACCGATTCAGAATGGCTCGAGAGAAGGTCTTTCCTGATTGGCTTTTTA-3′)and cloned into the AAV (titer for AAV particles containing TREM2-shRNA and vehicle-shRNA: 1 × 10^13^ vg/mL).

### Lateral ventricle stereotactic injection

**Fig. 1:**
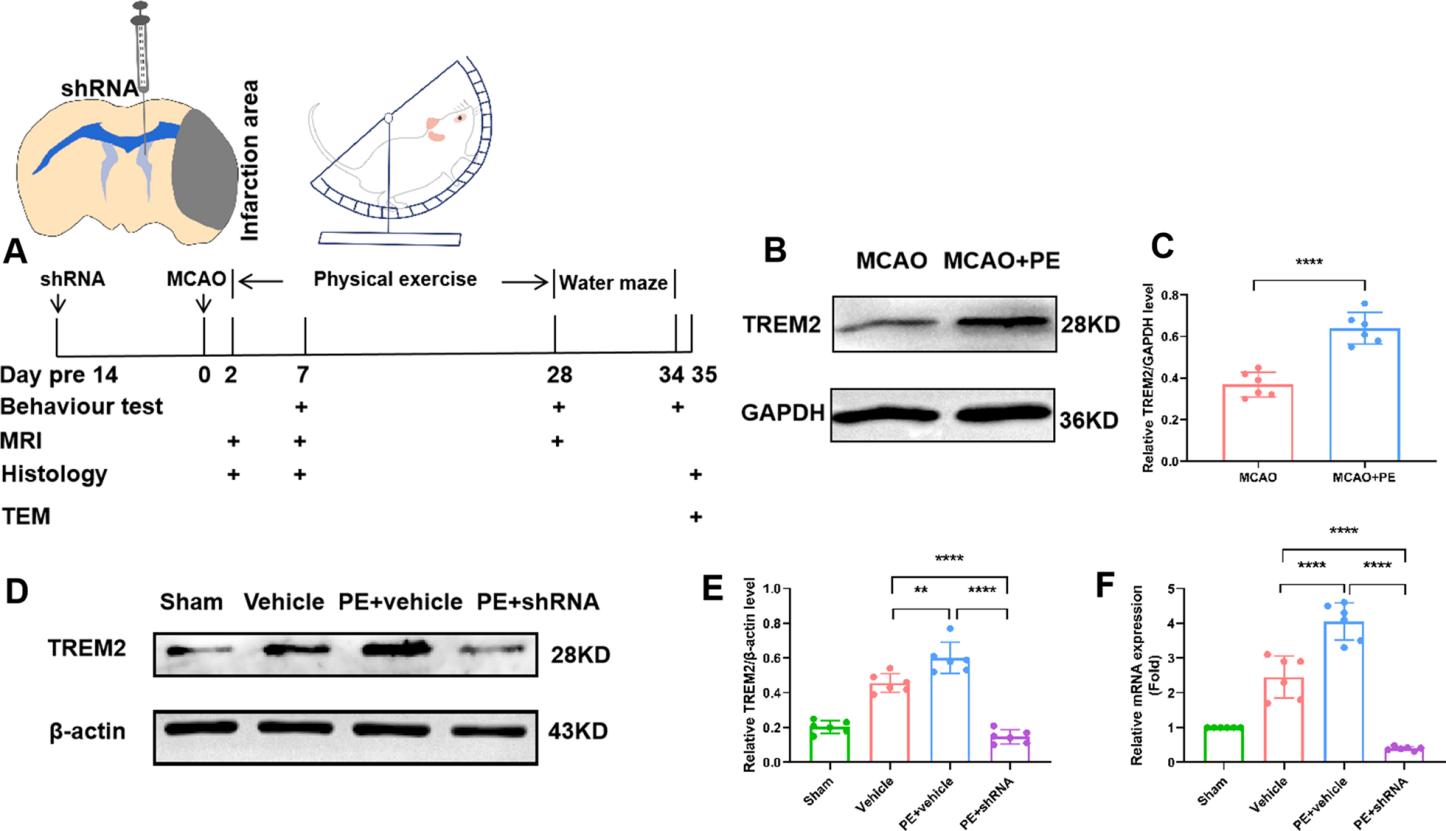
Physical exercise (PE) promotes TREM2 up-regulation in rats after stroke. **A** Schematic diagrams of experimental time points and the surgical procedure. **B**, **C** PE promotes TREM2 protein expression in rats with MCAO; **D**–**F** The TREM2 mRNA level and protein expression are up-regulated in the brain of rats 35 days after MCAO. Further, PE promotes TREM2 expression in rats with MCAO. While TREM2-shRNA injections abolish the PE-promoted up-regulation of TREM2. Data were analyzed by one-way analysis of variance followed by Tukey’s post hoc test. Each dataset is expressed as mean ± SD for n = 6. **P < 0.01; ****P < 0.0001

TREM2-shRNA (shRNA) or vehicle-shRNA (Vehicle) with AAV vector was injected into the left lateral ventricle of the rats 14 days before tMCAO by stereotactic injection (Fig. 1A); this was performed by technicians blinded to the experimental grouping. Before the operation, rats were anesthetized using 1% pentobarbital under spontaneous breathing and fixed on a stereotactic frame (RWD, Shenzhen, China). The liquid (5ul) was slowly injected into the lateral ventricles at 0.5 μL/min via a mini-pump (RWD, Shenzhen, China). Stereotactic coordinates of injection sites from bregma were: Posterior: 0.9 mm; mediolateral: 1.4 mm; and dorsoventral: 3.8 mm. The efficacy of AAV-mediated TREM2 silencing was established using quantitative real-time polymerase chain reaction (qRT-PCR) and Western blotting 14 days after injection. Based on our preliminary experiments, the TREM2 expression in rat brains was reduced to a relatively steady level 14 days after AAV particle injection.

### Injection of medication

A neutralizing antibody of activin-A (R&D systems, AF338) was intraperitoneally injected into the rats (Anti-activin A group, 0.084 mg/kg) after stroke once daily for 4 weeks.

### Physical exercise program

The PE program was performed as previously described [19, 20]. First, rats subjected to PE-treatment were placed into a programmable and motorized wheel apparatus (21 cm diameter, 40 cm length, Guangdong, China) for a moderate intensity running exercise intervention 48 h after MCAO. The initial running speed was set at 5 rev/min for 20 min, then increased to 7 revs/min on the 8th day, ten revs/min on the 15th day, and maintained this speed until behavioral testing. Rats were trained twice daily for six days per week. The sham and control groups had free access to water and food and were housed in a standard cage without PE training.

### Magnetic resonance imaging (MRI) scanning

MRI measurements were performed using a 7.0 Tesla horizontal-bore magnet (Bruker Biospin, PharmaScan70/16, US). During MRI measurements, a gas mixture of oxygen and isoflurane was used to maintain anesthesia, and a feedback-controlled electric blanket was used to keep the rectal temperature at 37 °C ± 0.5 °C. A tri-pilot imaging sequence was used to acquire reproducible positioning of the animal in the magnet at each MRI session. A complete set of MRI images was performed on the days 2, 7, and 28 after the operation.

Further, T2-weighted imaging (T2 × WI) with multiple spins or gradient echo sequences was performed. The parameters were as follows: field of view (FOV) = 35 × 35 mm^2^, image matrix = 128 × 128, TR/TE = 2500/33 ms, and slice thickness = 0.8 mm. All six echoes were acquired with an equal interval of echo time (TE) and under a similar readout gradient polarity in T2*WI. Diffusion tensor imaging (DTI) was performed with a spin-echo sequence. The parameters were as follows: TR/TE = 3500/38.5 ms, bandwidth = 1.05 kHz, slice thickness = 0.8 mm, FOV = 35 × 35 mm^2^, acquisition matrix = 128 × 128, and spatial resolution = 0.27 × 0.27 mm^2^. Directions were 6 and the b value was 1849.98 s/mm^2^.

### Modified neurological severity score (mNSS)

As previously described [21], mNSS, which determined by motor, sensory, balance and reflex tests was evaluated 7 and 28 days after tMCAO. The mNSS is assessed on a scale of 0–18, where “score 0” indicates normal function, and “score 18” indicates the maximal deficit.

### Morris water maze test

As previously described [10, 22], the spatial learning and memory of rats were evaluated by the Morris water maze (MWM) test from 29 to 34 days after the tMCAO operation. The training trials for rats to search the escape platform within 60 s were performed for five consecutive days. Rats were softly placed into the water with the head facing the wall. If the rat found the platform and stayed for 10 s, the accurate time it took was recorded. Meanwhile, the rat would be guided on the platform for 10 s if it failed to find the platform within 60 s, and was recorded as 60 s. Each rat was trained four times daily for five consecutive days. Thereafter, the escape platform was removed, then a 60-s spatial probe trial was conducted to evaluate the spatial memory retention of rats. The crossing times and the time spent in the target quadrant were recorded and analyzed.

### Novel object recognition test

The novel object recognition (NOR) test was performed as previously described to evaluate non-spatial memory [10, 23]. The rats were put into the apparatus a day before the test to adapt to the environment. The next day, the rats were successively placed in the centerline between two identical objects symmetrically in the open box and allowed to freely explore for 5 min to acquire memory. After 1 h, a novel object with different colors and shapes was used to replace one of the two objects. The time taken for animals to explore the familiar objects (F) and novel objects (N) were recorded. The differences among groups were compared, and the discrimination rate was calculated as N/(N + F) × 100%. 75% alcohol was used to eliminate the residual odor during the experiment.

### Quantitative real-time polymerase chain reaction

Brain tissues were quickly obtained from the ipsilateral white matter. qRT-PCR was used to measure mRNA expression level of *Trem2*, pro-inflammatory markers (*Inos, Cd86*), and anti-inflammatory & neuroprotective markers (C*d206, Arg1*) and was repeated in triplicate. We used ESscience reagent (ESscience, China) to extract total RNA. Complementary DNA synthesis was performed by ESscience cDNA synthesis kit (ESscience, China) following the manufacturer’s instructions. Primer sequences are listed in Table 1.

**Table 1:** Sequences of the primers used in the study

species	Gene	Primer sequences(5′–3′)
Rat	*Gapdh*-F	GGCAAGTTCAACGGCACAGTCA
	*Gapdh*-R	CGACATACTCAGCACCAGCATCAC
	*Cd86*-F	TGTTCAGTGTCTCCATCAGCCTATC
	*Cd86*-R	TTTGTAGACGACCAGCAGAAAGAGA
	*Inos*-F	AACGCTACACTTCCAACGCAACA
	*Inos*-R	AAGAACAATCCACAACTCGCTCCAA
	*Trem2*-F	TAGACTGTAGCCAAGATG
	*Trem2*-R	TAATTCTCCTCCTACTCAG
	*Cd206*-F	ACTGCGTGGTGATGAAAGG
	*Cd206*-R	TAACCCAGTGGTTGCTCACA
	*Arg-1*-F	CAGTGGCGTTGACCTTGTCTTGT
	*Arg-1*-R	TGGTTCTGTTCGGTTTGCTGTGAT

### Western blotting analysis

Total proteins were extracted using tissue protein extraction reagents. Total tissue proteins (20 µg/lane) were loaded and separated on sodium dodecyl sulfate polyacrylamide gels electrophoresis (SDS-PAGE) at 120 V for 90 min, followed by transferring to polyvinylidene fluoride (PVDF) membranes (Millipore, USA) at 300 mA for 2 h. After that, Membranes were incubated in 5% (w/v) milk for 1 h followed by incubation overnight at 4 °C with primary antibodies, including, rabbit anti-TREM2 (1:1000, PAB37053, Bioswamp, China), rabbit anti-TREM2 (1:1000, PA5-87933, ThermoFisher, USA), rabbit anti-MBP (1:1000, 78896S, Cell Signaling Technology, USA), Acvr2B (1:1000, ab128544 and ab180185, Abcam, UK), mouse anti-GAPDH (1:1000, T0004, Affinity, USA), rabbit anti-β-actin (1:1000, AF7018, Affinity, USA) overnight at 4 °C. After washes, the membranes were incubated with corresponding secondary antibodies for 1 h. The ECL western blotting detection kit (P007, ABP Biosciences, Japan) and an enhanced chemiluminescence system were used to examine protein bands. ImageJ software was used to analyze the gray value of protein bands.

### Tissue preparation for histochemistry

The sacrificed rats were transcardially perfused with 0.9% physiological saline and 4% paraformaldehyde at 4 °C. Then the brain samples were fixed with 4% paraformaldehyde at 4 °C for 24 h, followed by sequential immersion in 20% and 30% sucrose until they completely sunk. Successively, brain samples were embedded with an optimal cutting temperature compound (OCT) and then cut into coronal section (10 µm thick) on a cryostat (CM1900, Leica, Germany) used for immunofluorescence staining.

### Immunofluorescence staining

For immunofluorescence staining, brain sections were pretreated with citrate buffer for 5 min for antigen retrieval, then blocked with immunostaining blocking solution (P0102, Beyotime, China) at room temperature for 1 h. Then the sections were incubated with rabbit anti-Oligo2 (1:100, ab109186, Abcam, UK) overnight at 4 °C. For double immunofluorescence staining, the sections were incubated with mixtures of rabbit anti-IBA-1 (1:300, 019-19741, Wako, Japan) or mouse anti-IBA-1 (1:300, 016-26721, Wako, Japan) and rabbit anti-TMEM119 (1:100, NBP2-30551, Novus, USA), mouse anti-iNOS (1:100, ab210823, Abcam, UK), rabbit anti-CD206 (1:100, ab64693, Abcam, UK), rabbit anti-TREM2 (1:200, PA5-87933, ThermoFisher, USA), and mouse anti-activin-A (1:50, ab89307, Abcam, UK); and mixtures of rabbit anti-NG2 (1:100, AB5320, Millipore, USA) and mouse anti-activin-A (1:50, ab89307, Abcam, UK), Acvr2B (1:100, ab128544 and ab180185, Abcam, UK); The next day, sections were incubated with secondary antibodies after washing with phosphate-buffered solution (PBS). In addition, sections were incubated with Rabbit anti-BCAS1 FITC conjugated (1:50, C01226F, SAB, China). Finally, sections were mounted with a solution containing 4′,6-diamidino-2-phenylindole (DAPI). Fluorescence signals were observed under a microscope (BX63, Olympus).

### Luxol fast blue (LFB) staining

LFB staining was used to assess myelin content and integrity. Frozen sections were placed in 1:1 alcohol/chloroform overnight then hydrated back with 95% ethyl alcohol, followed by incubation with LFB solution at 60 °C overnight. Subsequently, slices were washed with 95% ethanol and dd H_2_O in sequence. Slices were placed in a lithium carbonate solution followed by 70% ethanol and dd H_2_O for 10 s for differentiation, and rinsed with 100% alcohol, and xylene, respectively, and then mounted with a neutral balsam. Slices were eventually observed under the microscope camera.

### Enzyme-linked immunosorbent assay (ELISA)

Activin-A concentrations from the ipsilateral white matter tissues around the infarction were measured using a rat activin-A ELISA kit (ab193733, Abcam, UK). All the experimental steps were performed following the manufacturer’s instructions.

### Transmission electron microscopy (TEM)

Rats were sacrificed as previously mentioned. The corpus callosum (C–C) and striatum were quickly dissected and fixed with a configured fixed solution at room temperature for 2 h. Samples were post-fixed with 1% osmium acid in 0.1 M PBS (pH 7.4) for 2 h, then sequentially dehydrated in different alcohol concentrations, and infiltrated with 1:1 mixture of acetone and Epon 812 embedding agent, and pure Epon 812 embedding agent overnight sequentially. Polymerization was performed at 60 °C for 48 h. Sections were counterstained with 2% uranic acid saturated aqueous solution and lead citrate, and eventually observed under a TEM. To compare the myelin thickness, the G-ratios (defined as axon diameter/fiber diameter) of the individual myelinated fibers were calculated.

### Statistical analysis

Power analyses were used to determine sample sizes for animal studies based on previous pilot studies and literature. Statistical analysis was conducted using SPSS20.0 software and data were presented as the mean ± standard deviation (SD). The data obtained from the MWM test were analyzed by repeated-measures analysis of variance (ANOVA). The data of NOR test, immunofluorescence staining, ELISA, and TEM were evaluated using one-way ANOVA with Bonferroni’s or Tukey’s multiple comparison test. Pearson correlation tests were used to analyze correlations between continuous data with normal distribution. The correlation tests between data with nonnormal distributions were performed using Spearman rank correlation analysis. The *P*-value < 0.05 was considered statistically significant.

## Results

### Physical exercise promotes *TREM2* up-regulation in rats after stroke

In our preliminary experiment, PE promoted the TREM2 protein expression in rats after MCAO (Fig. 1B, C). To verify the role of TREM2 in ischemic stroke, rats were randomly assigned to four groups. Consistent with the result of our preliminary experiment, TREM2 protein expression was up-regulated in rats subjected to MCAO 35 days after the operation, and PE further promoted TREM2 expression, while TREM2-shRNA injections abolished the PE-promoted up-regulation of TREM2, as demonstrated by western blotting and qRT-PCR analysis (Fig. 1D–F). Immunostaining revealed that compared to the vehicle and PE + shRNA rats, PE increased the intensities of TREM2 co-localized with Iba-1-positive cells both in corpus callosum and striatum (Additional file 1: Fig. S1A–D).

### Physical exercise promotes long-term recovery of neurological function after stroke

**Fig. 2:**
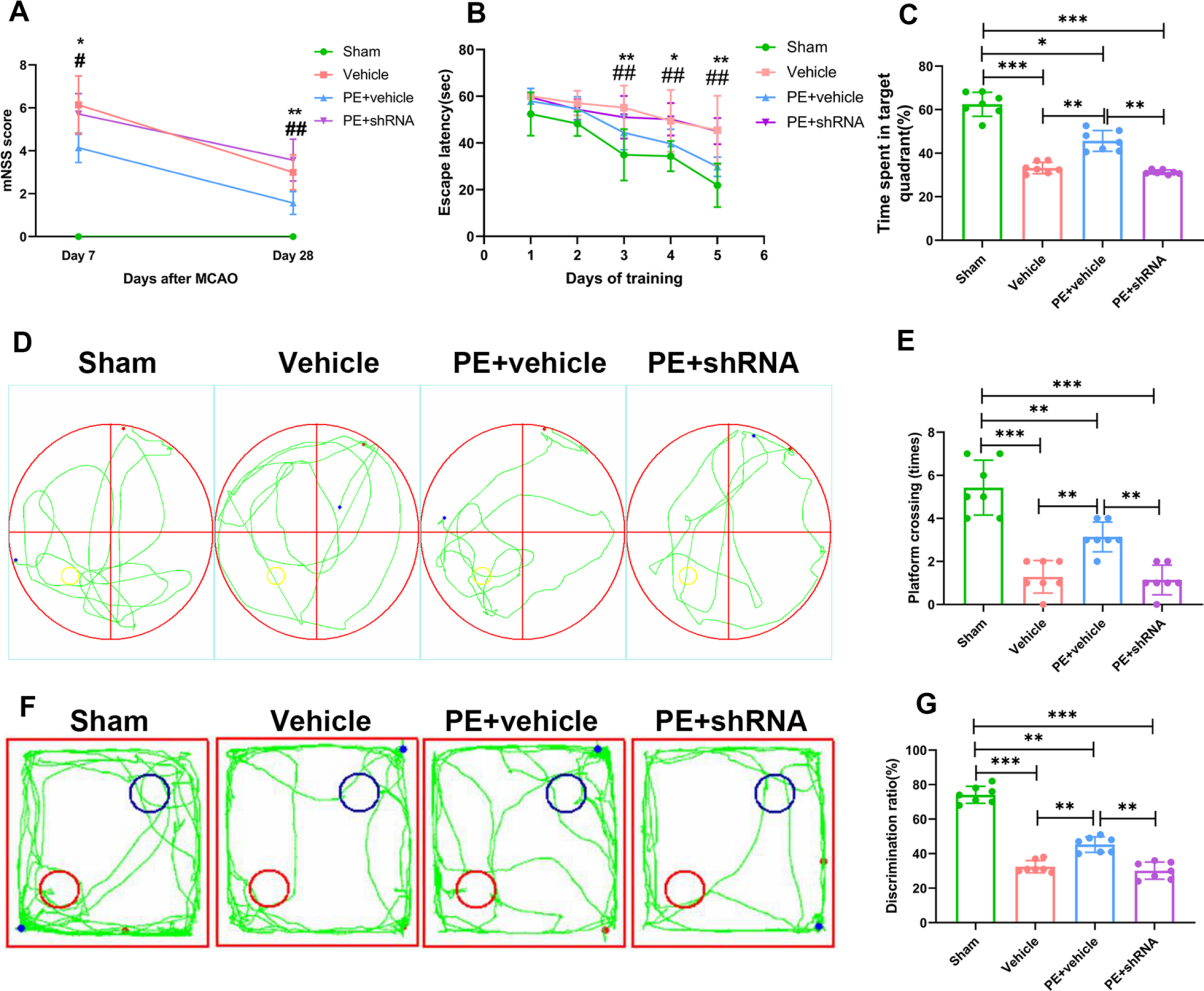
PE improves long-term neurological functions of rats after MCAO. **A** Analysis of modified neurological severity scores (mNSS). Vehicle vs. PE + vehicle, *P < 0.05; **P < 0.01; PE + vehicle vs. PE + shRNA, ^#^*P* < 0.05; ^##^*P* < 0.01. **B** Latency to find the platform during the training days of the Morris water maze (MWM) test. Vehicle vs. PE + vehicle, *P < 0.05; **P < 0.01; PE + vehicle vs. PE + shRNA, ^#^*P* < 0.05; ^##^*P* < 0.01. **C** Comparisons of the proportion of time spent in the target quadrant during probe trial of MWM test. **D** Representative images of the swim paths of rats during probe trial. **E** Comparisons of the crossing times during probe trial of MWM test. **F** Representative images of the moving paths of rats in each group in the novel objective recognition test; Purple circle: novel objects. **G** Comparisons of the discrimination ratio in various groups. Two-way repeated measures ANOVA or one-way ANOVA and Bonferroni post hoc tests. Each dataset is expressed as mean ± SD for n = 7. *P < 0.05; **P < 0.01; ***P < 0.001

Physical exercise promoted the recovery of motor function of rats after stroke, however, the effect of PE was weakened by TREM2-shRNA, as indicated by mNSS score in Fig. 2A. As shown in Fig. 2B–F, an impairment in learning abilities and memory was observed among MCAO rats. This was manifested by longer escape latency compared to the sham group on the 3rd to the 5th day of MWM training, the decreased time spent in the target quadrant during probe trial of MWM test, and decreased discrimination ratio in the NOR test. PE + vehicle shRNA (PE + vehicle) rats exhibited long-term improvements in these two tests compared to the vehicle-shRNA (Vehicle) rats and PE + TREM2-shRNA (PE + shRNA) rats. This was demonstrated by a reduction in the escape latency (Fig. 2B) and increased proportion of time spent in the target quadrant and the platform crossing times during probe trial (Fig. 2C–E), and an increased discrimination ratio (Fig. 2F and G). These findings indicate that PE improve the neurological function of rats with infarction via up-regulation of TREM2.

### Physical exercise promotes white matter integrity of rats after stroke by up-regulation of TREM2, and is associated with long-term recovery of neurological function

**Fig. 3:**
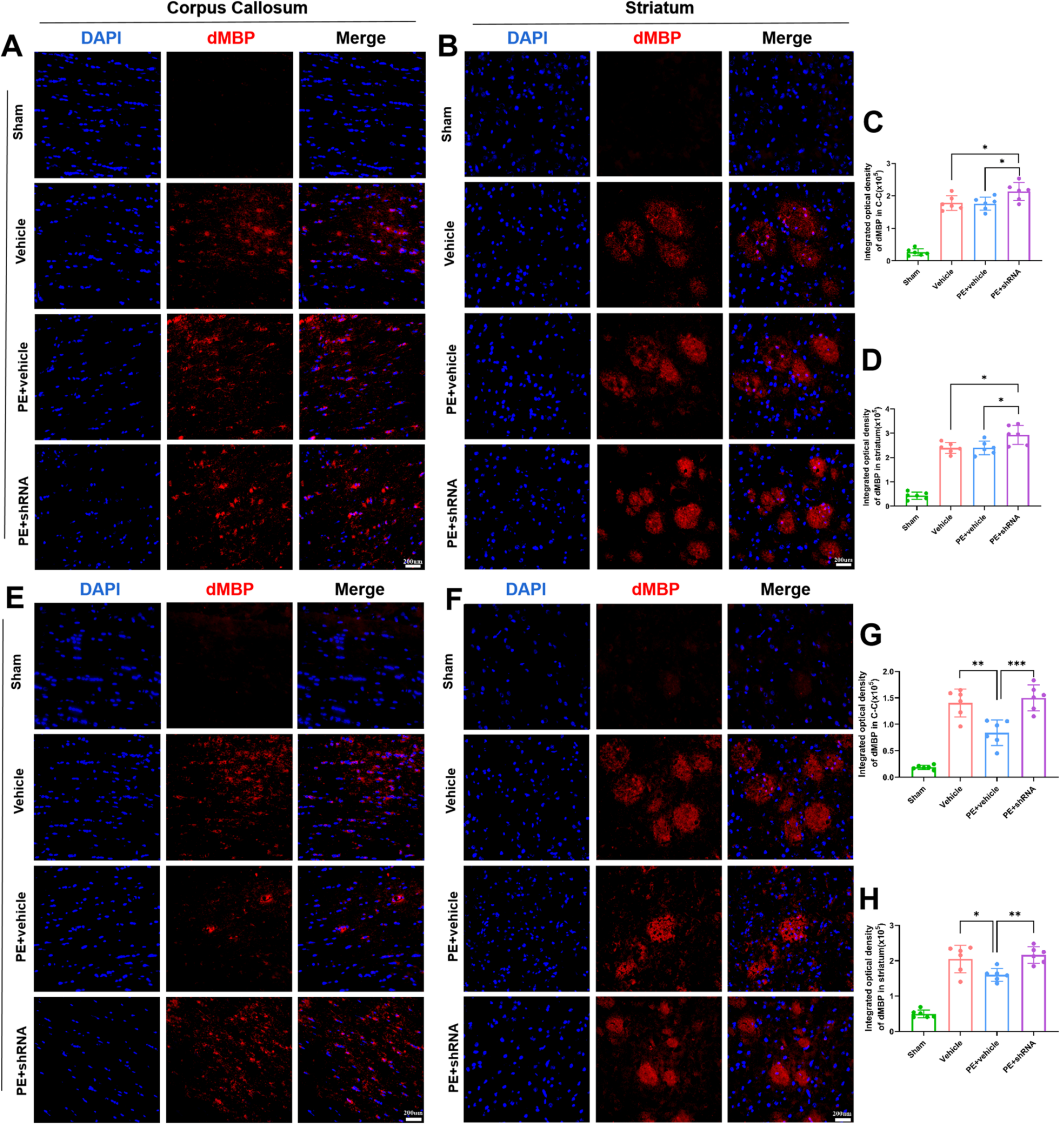
PE alleviates demyelination of rats subjected to MCAO. **A**, **B** Representative images of degraded myelin basic protein in corpus callosum (C–C) and striatum of rats prior to exercise (× 40 objective). **C**, **D** Comparisons of the integrated optical density in C–C and striatum of each group prior to exercise. **E**, **F** Representative images of dMBP in C–C and striatum of rats 35 days after stroke (× 40 objective). **G**, **H** Comparisons of the integrated optical density in C–C and striatumin of each group 35 days after stroke. One-way ANOVA and Bonferroni post hoc tests. Each dataset is expressed as mean ± SD for n = 6. *P < 0.05; **P < 0.01; ***P < 0.001

**Fig. 4:**
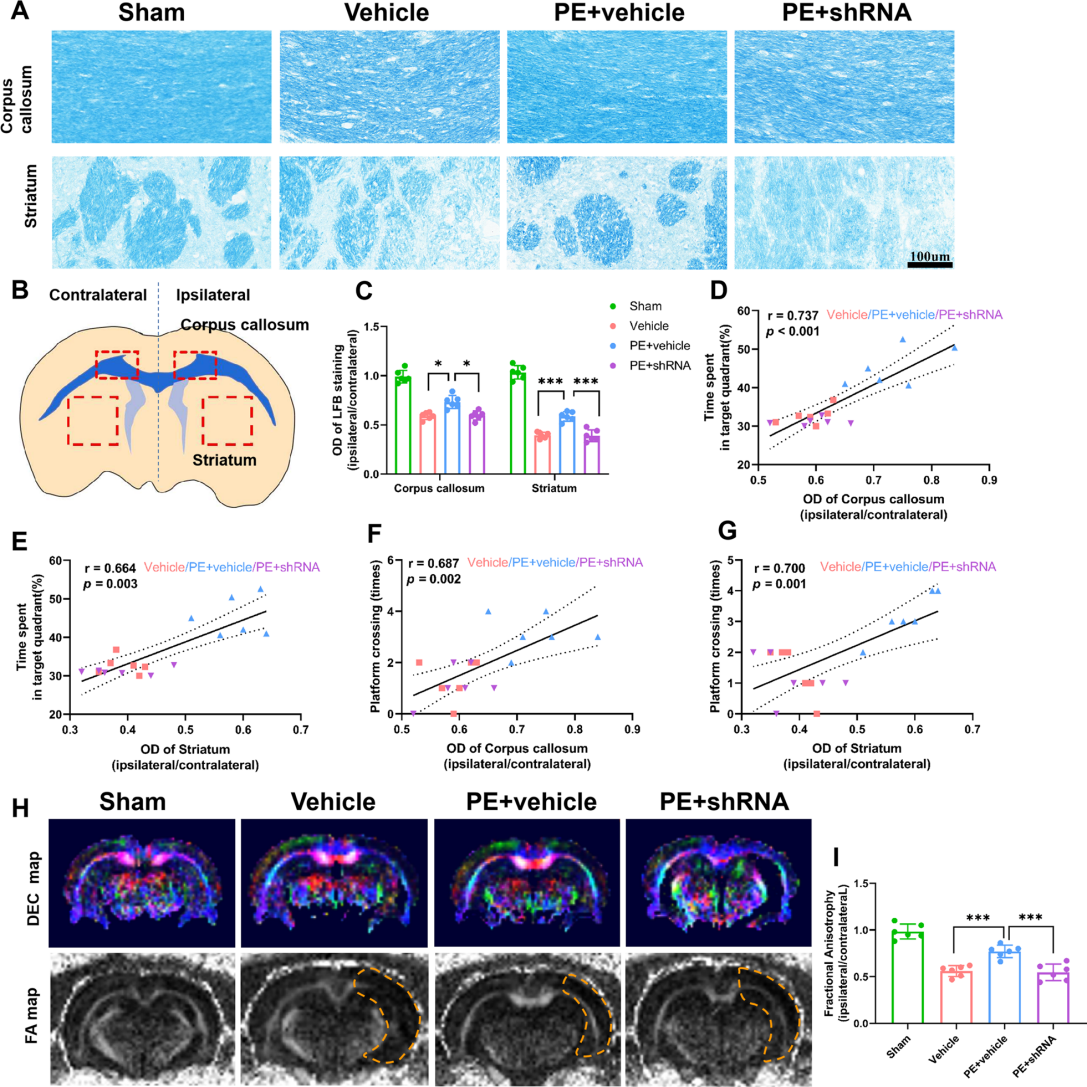
TREM2 is essential for PE-promoted white matter integrity of rats after stroke. **A** Representative LFB staining images of corpus callosum and striatum of rats in different groups. **B** Location of brain tissues used for staining or western blotting. **C** Comparisons of relative optical densities in the above mentioned structure. **D**–**G** Correlations between relative values of optical densities in corpus callosum, striatum and the increased proportion of time spent in the target quadrant as well as between relative values of optical densities in corpus callosum, striatum and platform crossing times. **H** Representative FA and DEC maps of rats on the 28th day after MCAO in different groups, dotted line indicates outline of the lesion border. **I** Comparison of the ratio of FA values in ipsilateral (lesioned) side relative to those in non-lesioned contralateral hemispheres. One-way ANOVA and Bonferroni post hoc tests. Data are expressed as mean ± SD for n = 6. *P < 0.05; ***P < 0.001

To confirm demyelination of rats after stroke, degraded myelin basic protein (dMBP) staining was performed. The images demonstrated that rats in Vehicle and PE + vehicle groups had similar demyelination before PE, while rats in PE + shRNA group showed more severe demyelination (Fig. 3A–D). PE significantly alleviated demyelination in ipsilateral C–C and striatum of rats 35 days after MCAO, compared to the rats in the Vehicle and PE + shRNA groups (Fig. 3E–H). Whole-brain in vivo DTI scans and LFB staining were performed 28/35 days after MCAO to evaluate the structural integrity of white matter after ischemic stroke and the promotion of PE on white matter remyelination. LFB staining revealed that the relative optical density of corpus callosum and striatum was significantly lower in rats subjected to MCAO 35 days after operation than sham rats. (Fig. 4A, C). Besides, the fractional anisotropy (FA) ratio (ipsilateral/contralateral) of corpus callosum-external capsule (EC) scanned by DTI in rats with MCAO demonstrated severe white matter injury, which was significantly alleviated in PE + vehicle-treated rats (Fig. 4H–I). However, the increased relative optical density and the FA ratio improvement were suppressed in TREM2-deficient rats with PE, indicating that TREM2-deficient inhibited the improvement of white matter integrity promoted by PE.

Further, correlations between white matter integrity and behavioral performance were analyzed in all three MCAO groups. There are significant positive correlations between myeline intensity (the relative values of optical density) in C–C, striatum, and the increased proportion of time spent in the target quadrant (Fig. 4D, E), and between myeline intensity in C–C and striatum and platform crossing times (Fig. 4F, G) 35 days after stroke. This demonstrates a correlation between improvement of white matter injury and long-term cognitive functional recovery.

### Physical exercise modulates microglial state via up-regulation of TREM2

**Fig. 5:**
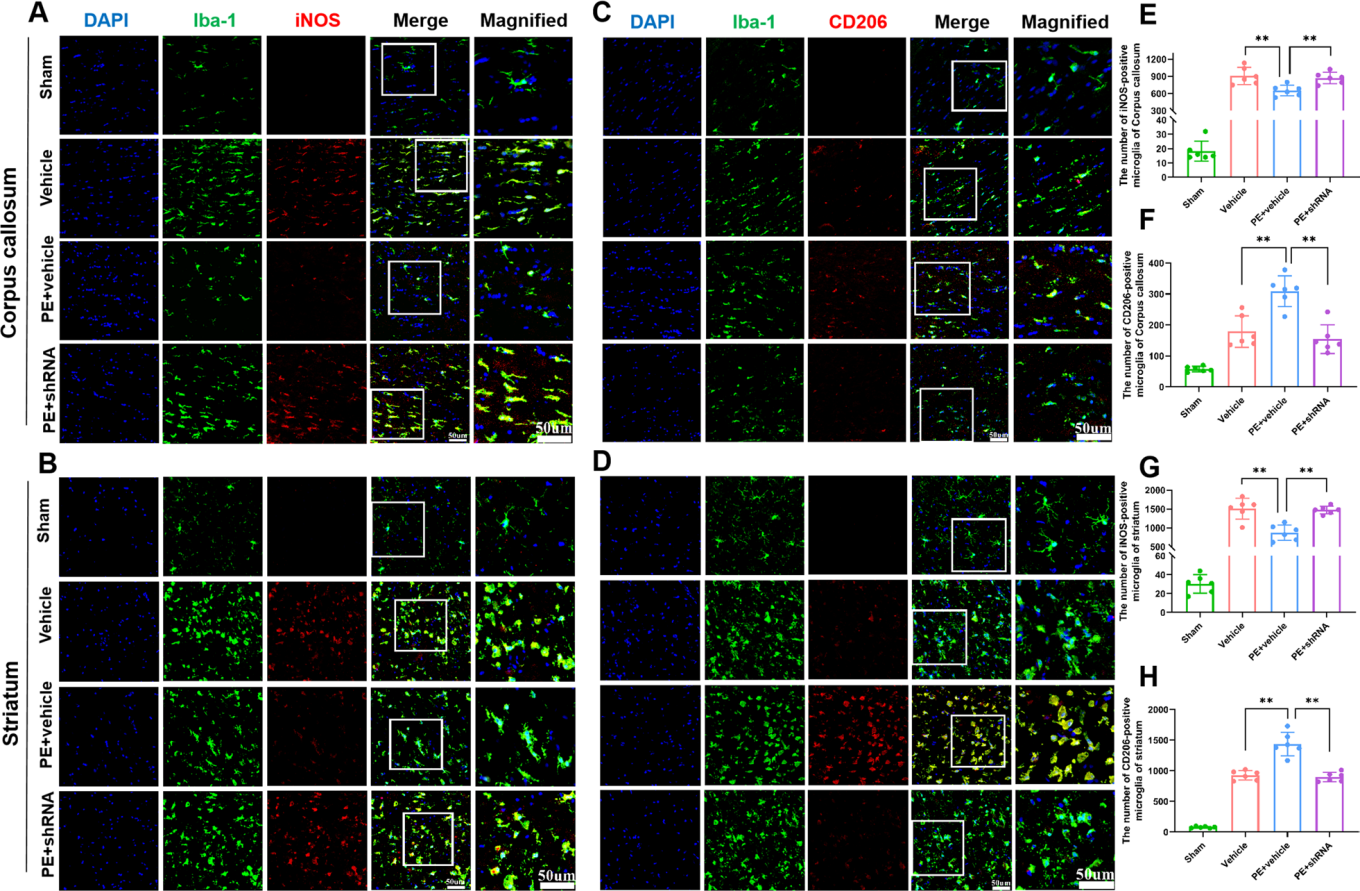
PE modulates microglial state via up-regulation of TREM2. **A**, **B** Representative images of iNOS^+^/Iba-1^+^ microglia in corpus callosum and striatum of each group (× 20 objective, zoomed in 2). **C**, **D** Representative images of CD206^+^/Iba-1^+^ microglia in corpus callosum and striatum of each group (× 20 objective, zoomed in 2). **E**, **F** Comparisons of the number of iNOS^+^/Iba-1^+^ microglia and CD206^+^/Iba-1^+^ microglia in corpus callosum of each group. **G**, **H**. Comparisons of the number of iNOS^+^/Iba-1^+^ microglia and CD206^+^/Iba-1^+^ microglia in striatum of each group. One-way ANOVA and Bonferroni post hoc tests. Each dataset is expressed as mean ± SD for n = 6. **P < 0.01

Since TREM2 is specifically expressed in CNS microglia, we determined whether PE modulated microglial state via TREM2 up-regulation. Among the three groups of rats subjected to MCAO in this study, over 85% of the Iba-1^+^ cells were also TMEM119^+^ in striatum around the ischemic zone (Additional file 1: Fig. S2), indicating that the contribution of infiltrating peripheral cells was comparatively minimal at this stage. In addition, unlike sham rats, Iba-1 immunostaining revealed that all the three groups of rats subjected to MCAO exhibited increased activation of Iba-1-positive cells in corpus callosum and striatum around the ischemic zone 7 days after MCAO (Fig. 5A–D). Intriguingly, PE reduced the number of iNOS^+^/Iba-1^+^ microglia (Fig. 5A and B), and increased the number of CD206^+^/Iba-1^+^ microglia (Fig. 5C and D) compared to vehicle and PE + shRNA rats. qRT-PCR analysis revealed comparable findings with regard to mRNA levels at both 7 and 35 days after stroke (Additional file 1: Fig. S3A-E). Collectively, these findings suggest that PE modulates microglial state after ischemia in sub-acute and remote phases in a TREM2-dependent manner..

### Physical exercise promotes activin-A expression via up-regulation of TREM2

Neuroprotective effect of microglia is critical for regenerative responses in the CNS by promoting oligodendrocyte lineage cell differentiation. Activin-A is an microglia-derived regenerative factor with the capacity for mediating oligodendrocyte differentiation.

**Fig. 6:**
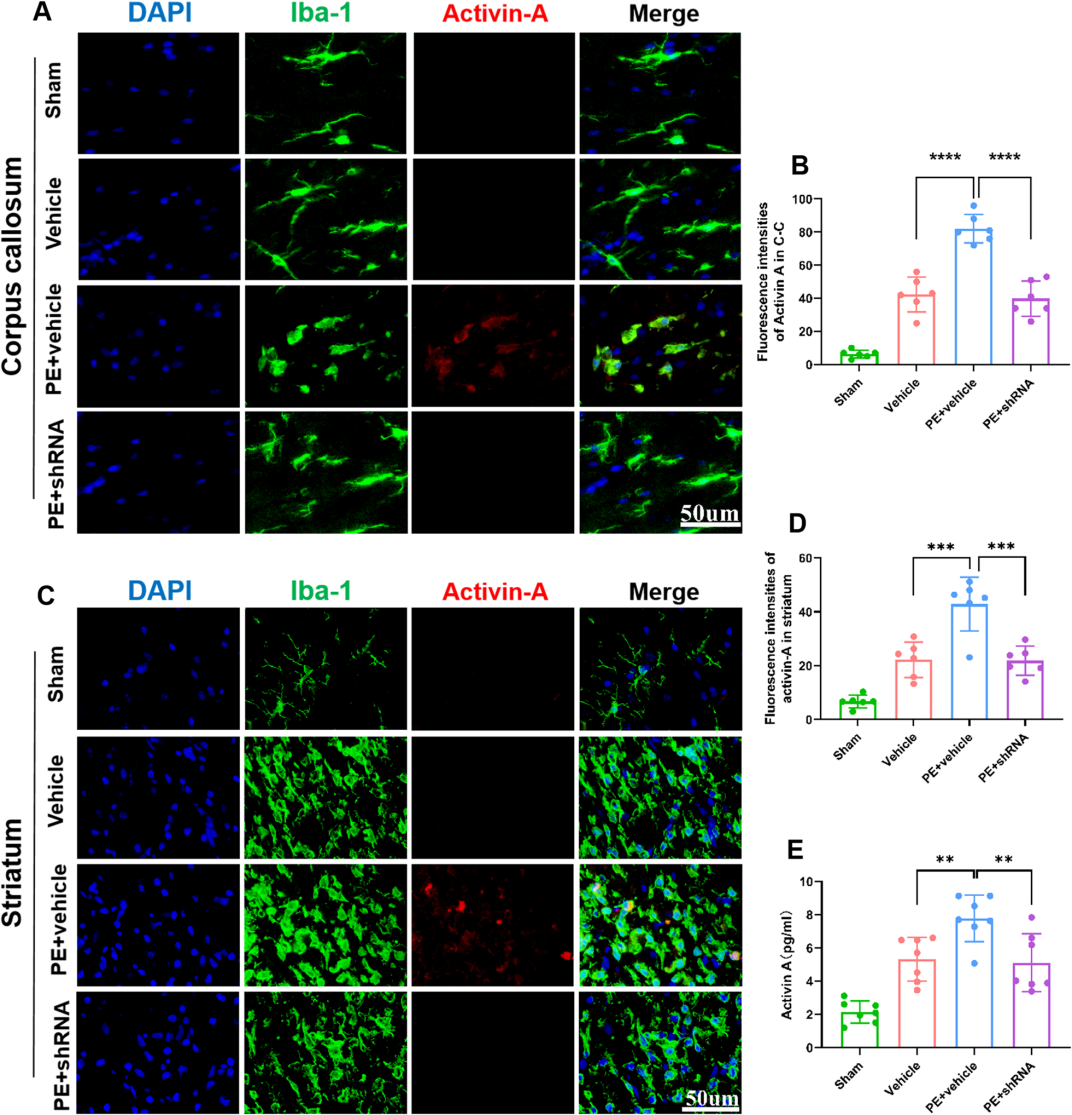
TREM2 is essential for PE-promoted up-regulation of activin-A. **A** Representative images of colocalization of activin-A and Iba-1^+^ microglia in corpus callosum of each group (× 40 objective, zoomed in 3). **B** Comparisons of fluorescence intensities of activin-A in corpus callosum of each group. **C** Representative images of colocalization of activin-A and Iba-1^+^ microglia in striatum of each group (× 40 objective, zoomed in 3). **D** Comparison of fluorescence intensities of activin-A in striatum of each group. **E** Comparisons of activin-A expression determined by ELISA of each group. One-way ANOVA and Bonferroni post hoc tests. Each data set is expressed as mean ± SD for n = 6. **P < 0.01; ***P < 0.001; ****p < 0.0001

**Fig. 7:**
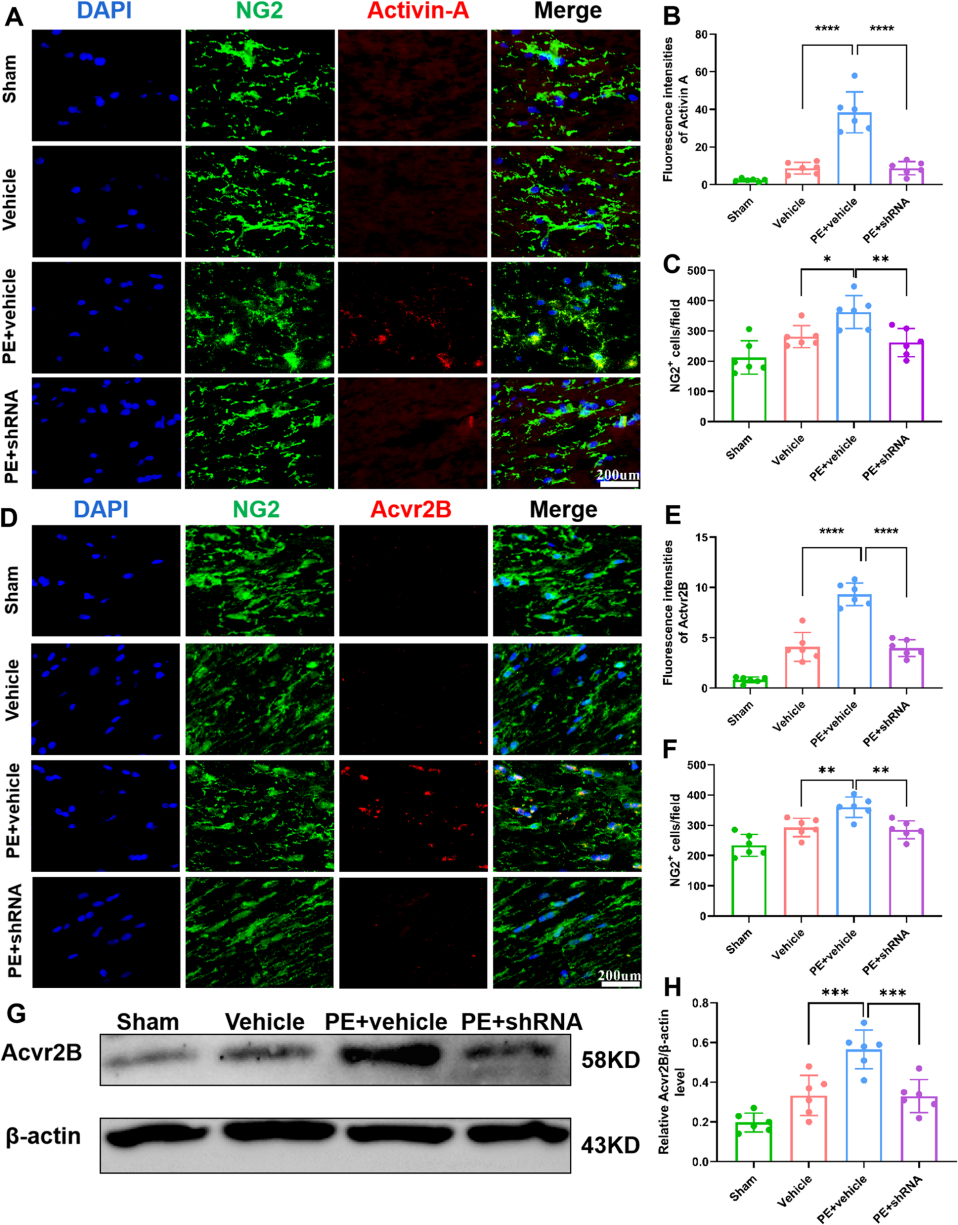
TREM2 is essential for PE-mediated up-regulation of activin-A/Acvr axis. **A** Representative images of colocalization of activin-A and NG2^+^ OPCs in corpus callosum of each group (× 40 objective, zoomed in 3). **B** Comparisons of fluorescence intensities of activin-A in each group. **C** Comparisons of NG2^+^ OPCs of each group. **D** Representative images of colocalization of acvr2B and NG2^+^ OPCs in corpus callosum of each group (× 40 objective, zoomed in 3). **E** Comparisons of Acvr2B fluorescence intensities in each group. **F** Comparisons of NG2^+^ OPCs of each group. **G** Chemiluminescence images of Acvr2B. **H** Comparisons of Acvr2B/β-actin ratio. One-way ANOVA and Bonferroni post hoc tests. Each dataset is expressed as mean ± SD for n = 6. *P < 0.05; **P < 0.01; ***p < 0.001; ****p < 0.0001

In this study, PE increased the immunofluence intensity of activin-A co-localized with Iba-1^+^ microglia (Fig. 6A–D) and NG2^+^ OPCs (Fig. 7A, B) in white matter, compared to the vehicle and PE + shRNA rats. That is, PE-regulated microglial state via up-regulation of TREM2 enhanced activin-A expression, which was also confirmed by ELISA (Fig. 6E). Additionally, expression of receptor that directly bind activin-A, Acvr2B was evaluated, where we noted an increase in NG2^+^ OPCs within remyelinating lesions in PE-treated rats (Fig. 7D, E), which was further confirmed by western blotting (Fig. 7G–H). These findings suggest that OPCs within remyelinating lesions can directly bind and respond to activin-A.

### Activin-A is essential for PE-induced oligodendrogenesis

**Fig. 8:**
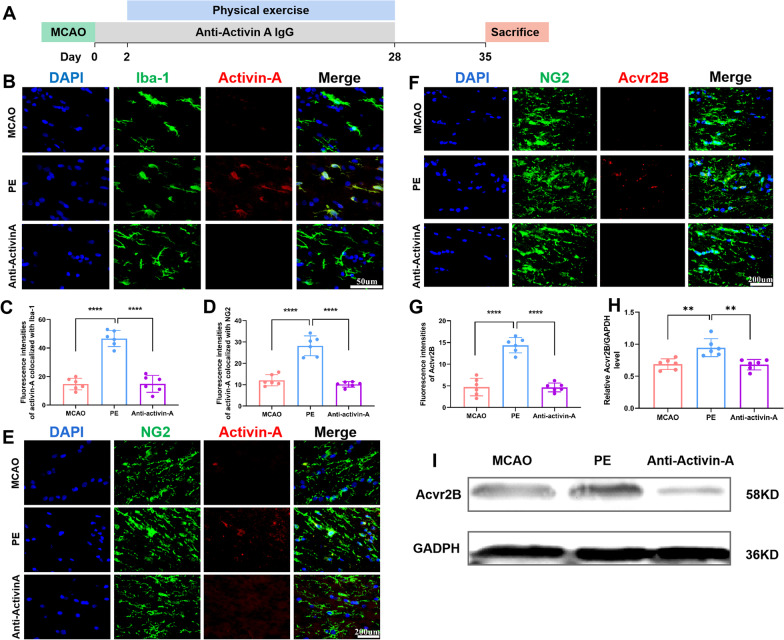
Expression of antivin-A/Acvr2B axis as determined by histological staining and western blotting. **A** Timepoint of the schematic design. **B** Representative images of colocalization of activin-A and Iba-1^+^ microglia in corpus callosum (× 40 objective, zoomed in 3). **C** Fluorescence intensities of activin-A co-localized with Iba-1^+^ microglia in each group. **D** Fluorescence intensities of activin-A co-localized with NG2^+^ OPCs in each group. **E** Representative images of colocalization of activin-A and NG2^+^ OPCs in corpus callosum (× 40 objective, zoomed in 3). **F** Representative images of colocalization of Acvr2B and NG2^+^ OPCs in corpus callosum (× 40 objective, zoomed in 3). **G** Fluorescence intensities of Acvr2B co-localized with NG2^+^ OPCs in each group. **H** Acvr2B/GAPDH ratio for the different groups. **I** Chemiluminescence images of Acvr2B and GADPH. One-way ANOVA and Bonferroni post hoc tests. Data are expressed as mean ± SD for n = 6. **P < 0.01; ****P < 0.0001

Further, we assessed whether activin-A is essential for PE-induced oligodendrogenesis after stroke. Consistent with the above results, we found that PE increased both the activin-A^+^ microglia and OPCs (Fig. 8B–E), while anti-activin-A antibody inhibited activin-A expression in white matter of stroke rats subjected to PE. Moreover, Acvr2B expressions were inhibited in PE-treated stroke rats after injection with anti-activin-A antibody (Fig. 8F–I).

**Fig. 9:**
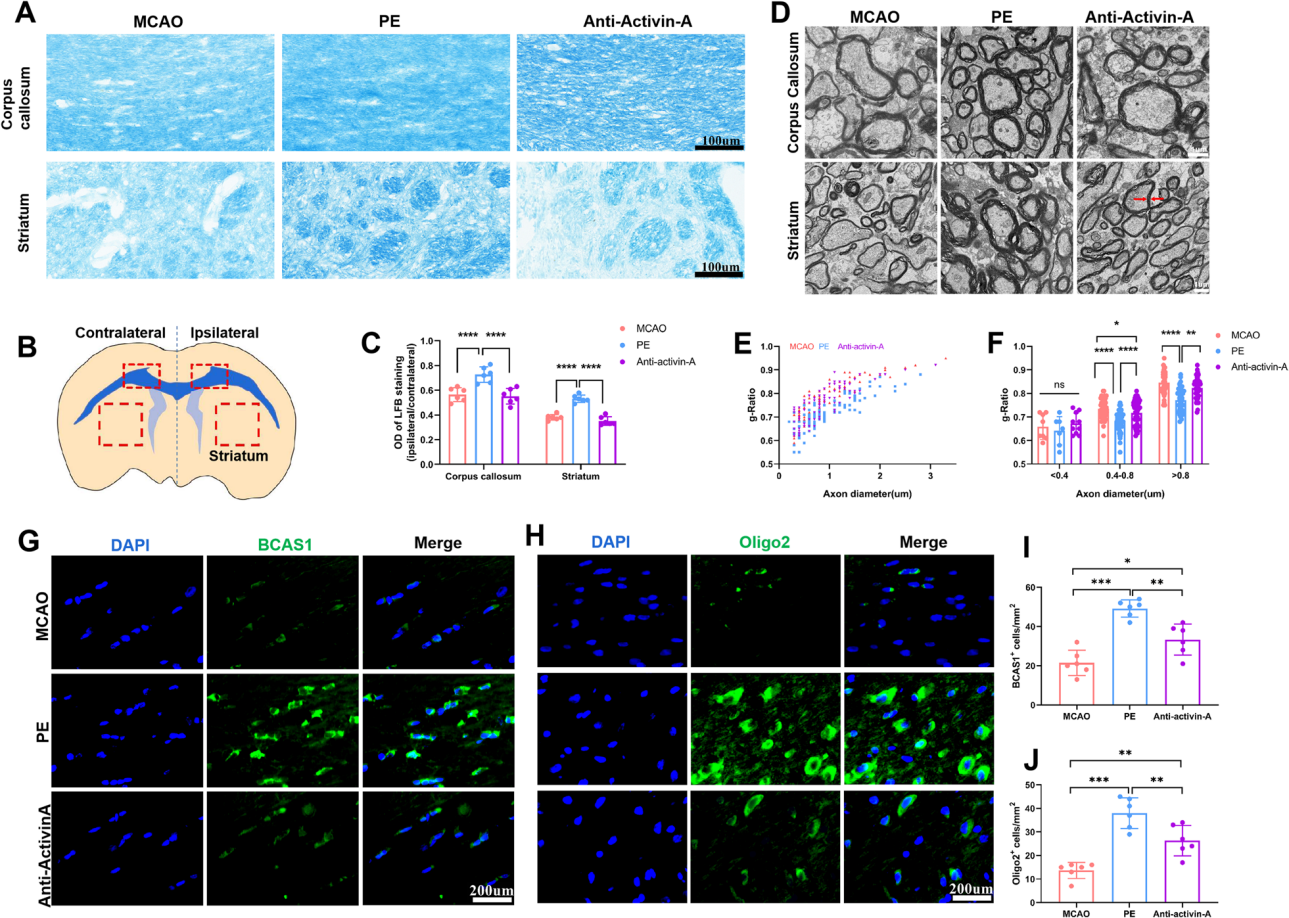
Activin-A is essential for PE-associated repair of white matter integrity and axonal remyelination. **A** Representative LFB staining images of corpus callosum and striatum of rats in different groups. **B** Location of the brain tissues used for staining or western blotting. **C** Relative optical densities in corpus callosum and striatum of rats in different groups. **D** Representative TEM images of myelin in ipsilateral corpus callosum and striatum. Red arrows refer to myelin thickness, bar = 1um; **E** Scatter plots of g-ratio in each group. **F** G-ratio of myelinated axons for each group of rats (n = 3 rats/group). **G** Representative images of BCAS1^+^ oligodendrocytes in various groups (× 40 objective, zoomed in 3). **H** Representative images of Oligo2^+^ oligodendrocytes in various groups (× 40 objective, zoomed in 3). **I** Comparison of the number of BCAS1^+^ oligodendrocytes for each group. **J** Comparison of the number of Oligo2^+^ oligodendrocytes for each group. One-way ANOVA and Bonferroni post hoc tests. Data are expressed as mean ± SD for n = 6. *P < 0.05; **P < 0.01; ***P < 0.001; ****P < 0.0001

LFB staining and transmission electron microscopy (TEM) were performed to evaluate the white matter integrity and axonal demyelination after ischemic stroke and promotion effects of PE. PE promoted white matter integrity, which is indicated by significant increases in relative optical density values in corpus callosum and striatum. However, anti-activin-A antibody rats showed decreased relative optical density values, indicating that suppression of activin-A weakened the PE-associated improvement of white matter integrity (Fig. 9A and C).

Furthermore, myelin thickness in the corpus callosum and striatum were measured using TEM (Fig. 9D–F). The results demonstrated greater reductions in axon diameters in MCAO and anti-activin-A antibody groups, relative to PE group, implying higher axonal degeneration. Scatter plots of g-ratio indicated significant differences in myelin thickness among three groups (average g-ratio: MCAO = 0.77 [n = 93 axons], PE = 0.72 [n = 96 axons], anti-activin-A = 0.76 [n = 96 axons]. Both the g-ratio of middle-sized (diameter = 400–800 nm) and large (diameter > 800 nm) axonal fibers were significantly increased in rats subjected to physical exercise, implying that PE may trigger remyelination after stroke. However, activin-A suppression weakened the PE-associated improvement of axonal myelination (Fig. 9D–F).

Then, we performed BCAS1 staining and Oligo2 staining to distinguish newly generated oligodendrocytes and active myelin formation. Both the number of BCAS1^+^ oligodendrocytes and Olig2^+^ oligodendrocytes were significantly elevated in PE rats, compared to MCAO and anti-activin-A rats, suggesting that microglia-associated activin-A in moderate PE stroke-rats promoted OPCs differentiation and oligodendrogenesis. However, anti-activin-A antibody suppressed PE-enhanced myelin formation and oligodendrogenesis, as indicated by decreased BCAS1^+^ and Oligo2^+^ oligodendrocytes (Fig. 9G–J).

## Discussion

Biologically, TREM2 is involved in switching microglial functional state, clearance of myelin fragments [10, 24], as well as promotion of neurological recovery. However, the role of physical exercise on TREM2 expressions after stroke and its subsequent effect have not been established. We found that moderate physical exercise promoted TREM2 expression, alleviated brain white matter injury and neurological function deficits of rats with MCAO. These effects were partly attributed to microglia-derived neuroprotection and activin-A/acvr axis-induced remyelination.

Repair of white matter injury is essential for neurological function recovery after stroke. White matter integrity can be improved through preservation of preexisting white matter structures or enhancement of white matter repair [2]. This study showed that enhancement of white matter repair is an important mechanism of action for physical exercise. Integrated optical intensities of dMBP staining shortly after stroke in Vehicle and PE + vehicle rats indicated similar degrees of initial white matter injury shortly after stroke. However, PE significantly promoted amelioration in white matter microstructure in latter stages of recovery, which is also shown by increased myelin thickness and relative optical values of white matters. These results indicate that white matter repair is attributed to a long-term benefit of PE, rather than preservation shortly after stroke. Moreover, PE upregulated activin-A/Acvr levels and increased the abundance of differentiating oligodendrocytes (Oligo2^+^) 35 days after stroke. This increase in oligodendrogenesis is important for effective remyelination. PE-mediated reconstitution of myelin structure promoted behavioral performances. Previously, it was established that physical exercise increased axonal myelination in mice, which was mainly attributed to mTOR-enhanced neuronal activities or promotion of proliferation and differentiation of OPCs mediated by mTOR [25]. Findings from a previous study by our group indicated that PE alleviated white matter injury by modulating microglial phenotype transformation [10]. However, the mechanisms through which PE promotes remyelination after stroke have not been fully established.

In this study, TREM2 levels were upregulated in rats with MCAO, consistent with findings from previous studies [15, 26]. In addition, physical exercise promoted TREM2 expression in MCAO rats up to 35 days after stroke. TREM2 is required for remyelination process [14], and disruption of the *TREM2/DAP12* pathway leads to demyelination- and axonal loss-associated neurodegeneration [15]. Therefore, we evaluated whether PE-promoted TREM2 enhanced remyelination and investigated the potential mechanism involved.

The findings showed that PE modulated microglial functional state and promoted remyelination after stroke by up-regulating TREM2. TREM2 is mainly expressed in the microglia in central nervous system, thus neural repair role of TREM2 is mainly attributed to microglia phagocytosis in previous studies [27, 28], but not microglial interaction with other cells. In fact, in addition to phagocytosis, some microglia-derived factors show significant protective roles in neural repair after stroke. For example, Activin-A plays an important role in microglia/macrophage-mediated oligodendrocyte differentiation during remyelination [18]. Notably, remyelination is mediated by newly formed mature oligodendrocytes, and OPCs are essential for formation of mature oligodendrocytes [3, 29]. Although the formation of new OPCs is a common process after ischemic brain injury, majority of the proliferating OPCs after cerebral infarction do not differentiate into mature functional oligodendrocytes [30–32]. This is mainly attributed to the deleterious environment in lesioned brains. Therefore, after stroke, recovery of white matter integrity through the natural process is significantly limited [33–35]. In this study, to assess newly generated or differentiating oligodendrocytes, we performed BCAS1 and Olig2 staining. The results indicated that both the abundance of BCAS1^+^ and Olig2^+^ oligodendrocytes were significantly increased in rats subjected to PE. That is to say, PE promotes remyelination after stroke via enhanced oligodendrocyte differentiation, as BCAS1 expression could identify newly generated oligodendrocytes [36]. Mechanistically, we found that PE induced OPCs differentiation benefit from microglia-drived anctivin-A and subsequently activating activin-A/Acvr signaling. Inhibition of activin-A abrogated PE-mediated alleviation of white matter injury after stroke. This effect was indicated by significantly decreased relative optical densities of myelin and axonal thickness in activin-A inhibitor-treated rats, compared to PE-treated rats. Axonal thickness (diameter 0.4 –0.8 μm) in activin-A inhibitor-treated rats was higher, relative to MCAO rats, indicating activin-A/Acvr signaling may be not an exclusive mechanism for PE-induced oligodendrogenesis in stroke, though Miron et al. reported that activin-A plays an important role in differentiation of OPCs [18]. In addition, Galectin-3 plays an important role in OPCs differentiation [37–39], However, its role in PE-promoted remyelination has not been fully elucidated.

In addition to its potential roles in microglia-mediated remyelination, PE promotes remyelination through other mechanisms. For instance, our previous study reported that PE promoted remyelination by switching astrocyte polarization towards the A2 phenotype and was involved in subsequent clearance of myelin debris [11]. The A1 phenotype is toxic for oligodendrocyte lineage cells while A2 polarization promotes the conversion of OPCs into mature oligodendrocytes, and is implicated in remyelination [40]. What is more, PE may promote white matter repair through enhanced neurogenesis and synaptic plasticity, as a beneficial feedback signal provided by relatively preserved structure of axon and electrical function could trigger OPC differentiation and remyelination process [41, 42]. That is to say, the effects of physical exercise maybe multi-target, but not limit in white matter, neuron or microglia, which partly account for the phenomenon that the functional data in this study shows no difference between PE + shRNA and Vehicle group. What is more, in this study, PE + shRNA rats were subjected to both TREM2 silence and long-term moderate physical exercise, while vehicle rats lack of such two interventions. Therefore, to some extent, it may be not sufficient to make a valid conclusion via direct comparisons between these two groups.

Although the protective effects of PE have been widely investigated, determination of optimal time window and intensity of PE is key. Initiation of exercise in the early stages after stroke may have harmful effects, whereas a timely initiated exercise can exert benefits after stroke [43]. Mild and moderate intensity exercise training results in more effective neuroprotection, compared to high intensity exercise training [19, 43, 44]. The exercise program used in this study was based on findings from our previous studies, which indicated that low and moderate intensity exercises initiated at 48 h post-stroke are an optimal combination for maximum functional recovery [19]. However, studies should explore the optimal program for application in clinical rehabilitation of patients.

Apart from the exercise program, both gender and age are important factors that influence the recovery of ischemic stroke. Considering that estrogen is a protective hormone for stroke, we used male rats in this study to eliminate its potential effects. Moreover, a series of studies reported that activated microglia produced inflammatory factors, impeded oligodendrocyte generation, and prolonged responses to CNS insults during aging [45, 46]. Therefore, we used young adult rats in this study to avoid potential effects of aging on microglial state and remyelination, and keep a homogenization of exercise. It also should be noted that the complexity of microglial phenotypes has been raised. These phenotypes are more of a spectrum of pro- and anti-inflammatory factors and the status of microglia in vivo may include a spectrum of different but overlapping functional phenotypes [47]. Hence, in this study, we concentrated more on the microglial state and its neuroprotective effect in the lesioned brain after stroke.

Despite white matter repair has been raised in post-stroke rehabilitation, currently, effective therapies targeted white matter injury need to be further investigated. Our findings indicated a novel mechanism of PE in post-stroke white matter repair. This novel effect is triggered by TREM2-dependent modulation of microglial neuroprotection. Furthermore, the results indicated that PE promotes oligodendrogenesis and remyelination via modulation of the activin-A/Acvr2B axis. Therefore, moderate PE is a potential therapeutic strategy for remyelination and recovery of neurological function after ischemic stroke. Although upregulation of TREM2 expression by PE plays an important role in white matter repair after stroke, optimal TREM2 level and duration of its upregulation, as well as its role in inhibiting neuronal apoptosis should be elucidated through further studies.

## Conclusions

The present study reveals a novel regenerative role of PE in white matter damage after stroke, which is mediated by upregulation of TREM2 and microglia-derived factor for oligodendrocytes regeneration. PE is an effective therapeutic approach for improving white matter integrity and alleviating neurological function deficits after ischemic stroke.

## Supplementary Information

**Additional file 1.** Supplemental data file.

## Data Availability

All data generated or analyzed during this study are included in this published article (and its additional information files).

## Abbreviations

TREM2: Triggering receptors expressed on myeloid cells 2
PE: Physical exercise
tMCAO: Transient middle cerebral artery occlusion
OPCs: Oligodendrocyte precursor cells
MWM: Morris water maze
NOR: Novel object recognition
Iba-1: Ionized calcium-binding adapter molecule 1
iNOS: Inducible NO synthase
LFB: Luxol fast blue
DTI: Diffusion tensor imaging
C–C: Corpus callosum
EC: External capsule
