# Supplementary material for: TREM2 mediates physical exercise-promoted neural functional recovery in rats with ischemic stroke via microglia-promoted white matter repair
Source: J Neuroinflammation. 2023 Feb 25;20:50. doi: 10.1186/s12974-023-02741-w (PMC9960657; doi:10.1186/s12974-023-02741-w)
Supplement: Supplementary file 1 — Additional file 1. Supplemental data file. [file 12974_2023_2741_MOESM1_ESM.docx]

**
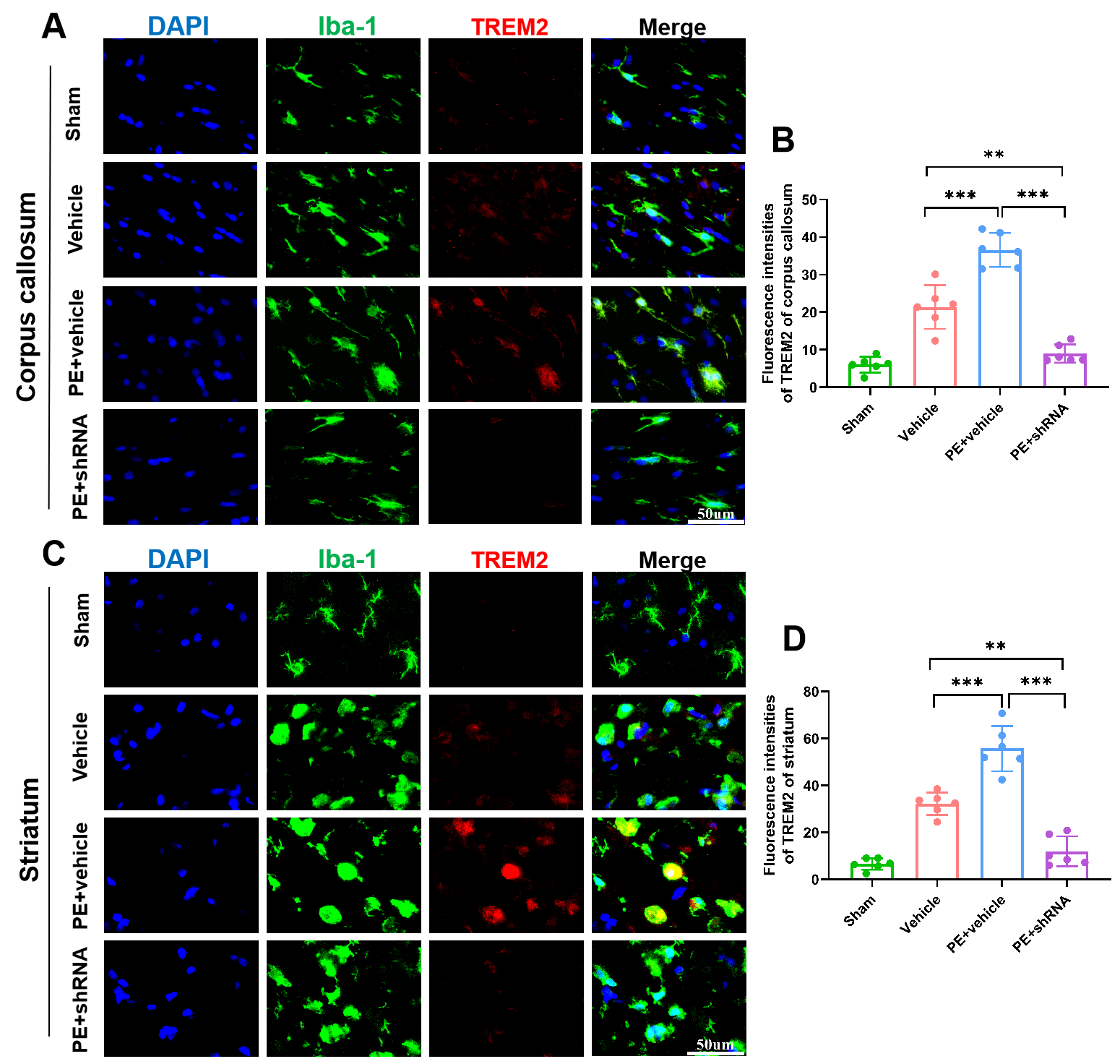
**

**Supplemental Figure 1. Physical exercise (PE) promotes TREM2 up-regulation in rats after stroke.** (A) Representative images of TREM2-positive microglia in corpus callosum of each group (×40 objective, zoomed in 3). (B) Comparisons of the intensities of TREM2 co-localized with microglia in corpus callosum of each group. (C) Representative images of TREM2-positive microglia in striatum of each group (×40 objective, zoomed in 3). (D) Comparisons of the intensities of TREM2 co-localized with microglia in striatum of each group. One-way ANOVA and Bonferroni post hoc tests. Each dataset is expressed as mean ± SD for n=6. ***P* < 0.01; ****P* < 0.001; *****P* < 0.0001.

**
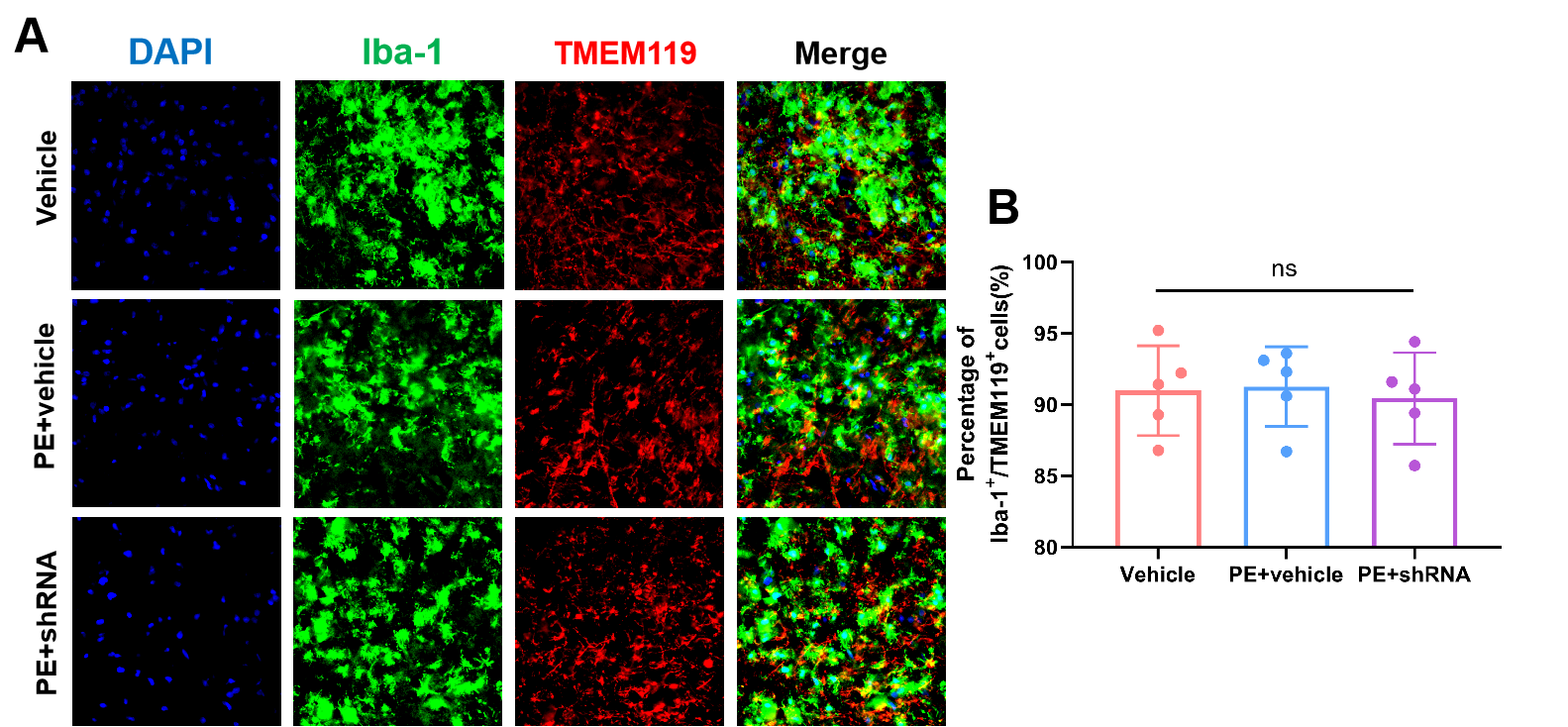
Supplemental Figure 2. Characterizing the Iba-1+ population cells by immunostaining with a more specific microglia marker (TMEM119).** (A) Representative images of Iba-1^+^/TMEM119^+^ microglia in striatum of each group (×40 objective, zoomed in 2). (B) The percentage of Iba-1+ cells colocalized with TMEM119 in striatum of each group. Each dataset is expressed as mean ± SD for n = 5.


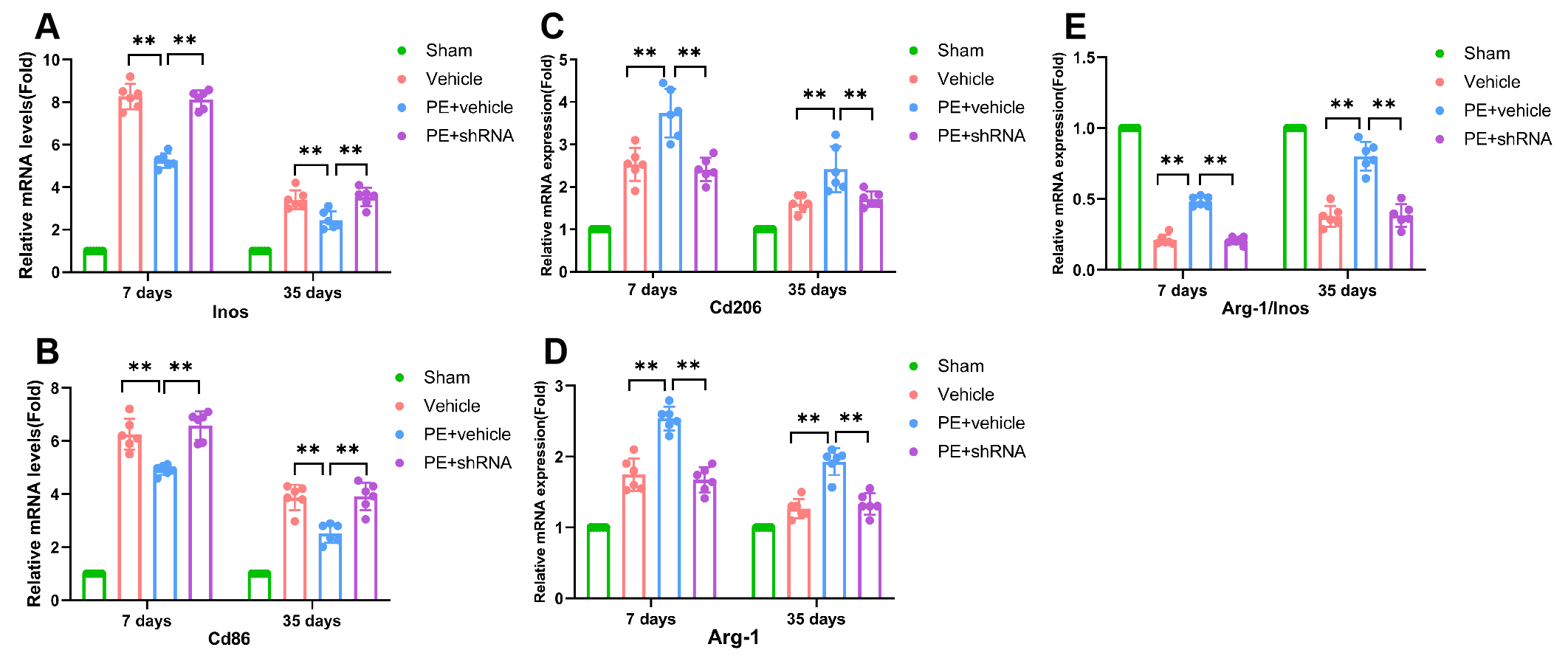


**Supplemental Figure 3. PE Promotes up-regulation of M2 microglia markers in a TREM2-dependent manner.** (A-B) mRNA levels of M1 (*Inos, Cd86*) microglia markers were decreased in PE + vehicle rats both in 7 days and 35 days after stroke meassured by qRT-PCR. (C-D) mRNA levels of M2 (C*d206, Arg-1*) microglia markers were increased in PE + vehicle rats both in 7 days and 35 days after stroke. While *Trem2*-shRNA abolished the role of PE. (E) The ratios of *Arg-1* to *Inos* mRNA level were increased in PE + vehicle rats.
